# An extensive benchmark study on biomedical text generation and mining with ChatGPT

**DOI:** 10.1093/bioinformatics/btad557

**Published:** 2023-09-08

**Authors:** Qijie Chen, Haotong Sun, Haoyang Liu, Yinghui Jiang, Ting Ran, Xurui Jin, Xianglu Xiao, Zhimin Lin, Hongming Chen, Zhangmin Niu

**Affiliations:** AIDD, Mindrank AI Ltd, Zhejiang 310000, China; AIDD, Mindrank AI Ltd, Zhejiang 310000, China; College of Life Sciences, Nankai University, Tianjin 300071, China; Guangzhou Laboratory, GuangDong 510005, China; AIDD, Mindrank AI Ltd, Zhejiang 310000, China; Guangzhou Laboratory, GuangDong 510005, China; AIDD, Mindrank AI Ltd, Zhejiang 310000, China; AIDD, Mindrank AI Ltd, Zhejiang 310000, China; AIDD, Mindrank AI Ltd, Zhejiang 310000, China; Guangzhou Laboratory, GuangDong 510005, China; AIDD, Mindrank AI Ltd, Zhejiang 310000, China; National Heart and Lung Institute, Imperial College London, London, United Kingdom

## Abstract

**Motivation:**

In recent years, the development of natural language process (NLP) technologies and deep learning hardware has led to significant improvement in large language models (LLMs). The ChatGPT, the state-of-the-art LLM built on GPT-3.5 and GPT-4, shows excellent capabilities in general language understanding and reasoning. Researchers also tested the GPTs on a variety of NLP-related tasks and benchmarks and got excellent results. With exciting performance on daily chat, researchers began to explore the capacity of ChatGPT on expertise that requires professional education for human and we are interested in the biomedical domain.

**Results:**

To evaluate the performance of ChatGPT on biomedical-related tasks, this article presents a comprehensive benchmark study on the use of ChatGPT for biomedical corpus, including article abstracts, clinical trials description, biomedical questions, and so on. Typical NLP tasks like named entity recognization, relation extraction, sentence similarity, question and answering, and document classification are included. Overall, ChatGPT got a BLURB score of 58.50 while the state-of-the-art model had a score of 84.30. Through a series of experiments, we demonstrated the effectiveness and versatility of ChatGPT in biomedical text understanding, reasoning and generation, and the limitation of ChatGPT build on GPT-3.5.

**Availability and implementation:**

All the datasets are available from BLURB benchmark https://microsoft.github.io/BLURB/index.html. The prompts are described in the article.

## 1 Introduction

In recent years, there has been a tremendous growth in the field of natural language processing (NLP) and machine learning. One of the most significant advancements in NLP is the development of large language models such as Generative Pretrained Transformer (GPT) (Radford *et al.* 2018, 2019, Brown *et al.* 2020) and its various variants, which have shown remarkable performance in a number of language tasks. Usually, GPT models were initially pretrained on massive text data and then fine-tuned on specific downstream tasks to generate human-like languages.

In the domain of biomedical text mining, NLP techniques have demonstrated the potential to revolutionize research and clinical practice. However, the complexity of biomedical language and the vast amount of data size still make it a challenging task to develop robust models for text generation and mining. In this article, we present a comprehensive benchmark study on evaluating the performance of ChatGPT model (Ouyang et al. 2022), a large-scale GPT-based language model, for biomedical text generation and mining.

The article is organized as follows: First, we will provide an overview of the related work in biomedical text mining and highlight the strength and limitations of current approaches; Second, the ChatGPT model and its applications in NLP will be described. Third, we will discuss the benchmarking and experimental protocols conducted in this study; Finally, we will present the performance of ChatGPT in various biomedical text generation and mining tasks along with other baseline biomedical NLP models and discuss the potential applications and future directions of ChatGPT in biomedical research and clinical practice.

Overall, this article aims to contribute to the growing body of research in the field of biomedical NLP by providing a comprehensive evaluation of ChatGPT model on biomedical text generation and mining. By comparing the performance of ChatGPT with other SOTA biomedical models on several biomedical-related NLP benchmark sets, we hope to provide the pros and cons of ChatGPT model in dealing with biomedical-related tasks, which may inspire further development of more advanced NLP models for biomedical data analysis.

### 1.1 Background and related work

In recent years, natural language processing (NLP) techniques have gained significant attention in the biomedical domain due to the vast amount of textual data generated by scientific publications, electronic health records, social media, etc. Biomedical text mining, a sub-field of NLP, aims to extract, analyse, and summarize useful information, and derive insightful knowledge from either structured or unstructured biomedical texts. Usually, extracting knowledge from biomedical text requires substantial human effort and is time-consuming. Thus, automated text generation and mining techniques can greatly assist researchers via extracting or deriving valuable insights from the available big data in biomedical literatures.

Recently, one of the most promising advances in NLP field is the development of so called large-scale language models (LLMs) using hundreds of billions of parameters and trained on gigabytes of text (Brown *et al.* 2020, Ouyang *et al.* 2022). These models have been shown to achieve state-of-the-art (SOTA) performance in several NLP tasks, including text generation, question and answering (QA), and text summarization. The capability of these models to generate coherent and contextually relevant text makes them ideal candidates for biomedical text generation and mining. By identifying critical data points for clinical trials and drug discovery, LLMs can assist in advancing the creation of new drugs and treatment approaches.

Several studies have demonstrated the potential of these language models in biomedical text mining. For instance, BioLinkBERT was an LM pretraining method that leverages links between biomedical documents. SciFive (Phan *et al.* 2021) applied a domain-specific T5 model (Raffel *et al.* 2020) that has been pretrained on large biomedical corpora.

Moreover, *pretrain, prompt, and predict* (Liu *et al.* 2023) is an emerging paradigm for applying LLMs to new problems without fine-tuning the weights on the task. Prompt-based learning involves enhancing the problem statement with specific instructions so that the model’s response to the prompt results in a solution. This methodology enables LLMs to learn from a limited set of examples, referred to as few-shot learning, which are integrated into the prompts themselves (Brown et al. 2020). ChatGPT (Ouyang *et al.* 2022) has garnered enormous attention due to its remarkable success in instruction understanding and human-like response generation. According to recent research, the ChatGPT language model created by OpenAI has shown promising results in performing at par with humans on MBA exams conducted by the Wharton Business School (Rosenblatt 2023). This indicates that AI language models like ChatGPT have the potential to compete with human and could be utilized to assist professionals (Baidoo-Anu and Owusu Ansah 2023, Choi *et al.* 2023). Also, their impressive performance on diverse NLP tasks, coupled with their ability to generalize to unfamiliar tasks, highlights their potential as a versatile solution for a variety of challenges in natural language understanding, text generation, and conversational AI.

While these studies have demonstrated the potential of LLMs in biomedical text mining, there is still a lack of comprehensive evaluation of LLMs on broad biomedical tasks. This study aims to provide a large-scale study of the latest ChatGPT model in biomedical text generation and mining. We investigated the performance of ChatGPT in several biomedical NLP tasks, including entity recognition, paragraph summarization, answer generation, etc. We also explored the possibility of using ChatGPT to assist researchers in extracting useful knowledge from the available biomedical data.

### 1.2 ChatGPT for biomedical NLP

The volume of biomedical literature has significantly expanded in recent years, leading to an urgent need for robust text mining tools for biomedical application. Numerous studies have shown that pretrained language model can help accelerate the progress of general biomedical NLP applications.

A common workflow for training domain specific language model is to pretrain models on large general datasets to learn general features of languages and then fine-tune on more focused domain specific data. Large models, e.g. BERT-based or GPT-based models, were first pretrained with huge amount of text data either supervisedly, semi-supervisedly, or unsupervisedly. The pretrained models offer representation, or in another word, featurization for input text, which is regarded as general understanding of the model for the input sentences. Then for any downstream task, the pretrained model fine-tuned with a relatively small domain specific train set in a supervised pattern. In some studies, the parameters of the pretrained model may also be frozen. The prediction head gives a desired output that can be utilized to evaluate the model performance. ChatGPT is a generative model based on GPT-3.5 and fine-tuned to accomplish text generating tasks. As the exact model structure and parameters are not released by OpenAI yet, it is impossible to directly fine-tune the model toward user supplied data. However, it has been shown that ChatGPT can achieve human-like dialogue results through chatting with specifically engineered prompts. Here, we employed prompt engineering method to engage ChatGPT model in biomedical related NLP tasks and then evaluate its performance. In most of cases, the ChatGPT model was challenged in a zero-shot or few-shot manner (as part of the prompt).

The design of the prompt is crucial for the output of ChatGPT. In general, the prompt should at least consist of a body of background context, an instruction part telling ChatGPT what’s the task supposed to be done, and a constrain part for formatting the output and content. For instance in a yes/no QA task, ChatGPT should be told to ’answer in a simple yes or no’ so that we can obtain structured results and calculate performance metrics. But there are still cases that output of ChatGPT does not obey the constrains, e.g. supplying reasons after a “yes” for a QA task or answering entities that does not exist in the text for a named entity recognization (NER) task. To deal with these exceptions, we designed formulas to automatically check the output. For exceptions, we would send for correction, pointing out the problem, emphasizing the format and requesting an answer again. This leads to multiple rounds of question and answering. Here is an example:

Prompt: *Question: In clinical trials, does the H3R antagonist CEP-26401 have a positive effect on cognition? Answer with yes or no.*

Answer of ChatGPT: *Clinical proof of concept for CEP-26401 in cognition enhancement has not been reported yet. Therefore, I cannot answer with “yes” or “no” as there is no conclusive evidence from clinical trials.*

The answer is neither “yes” nor “no”. A following prompt is triggered: *Your answer is last answer, but you are supposed to answer with yes or no. Answer the former question again.*

**Figure 1.**
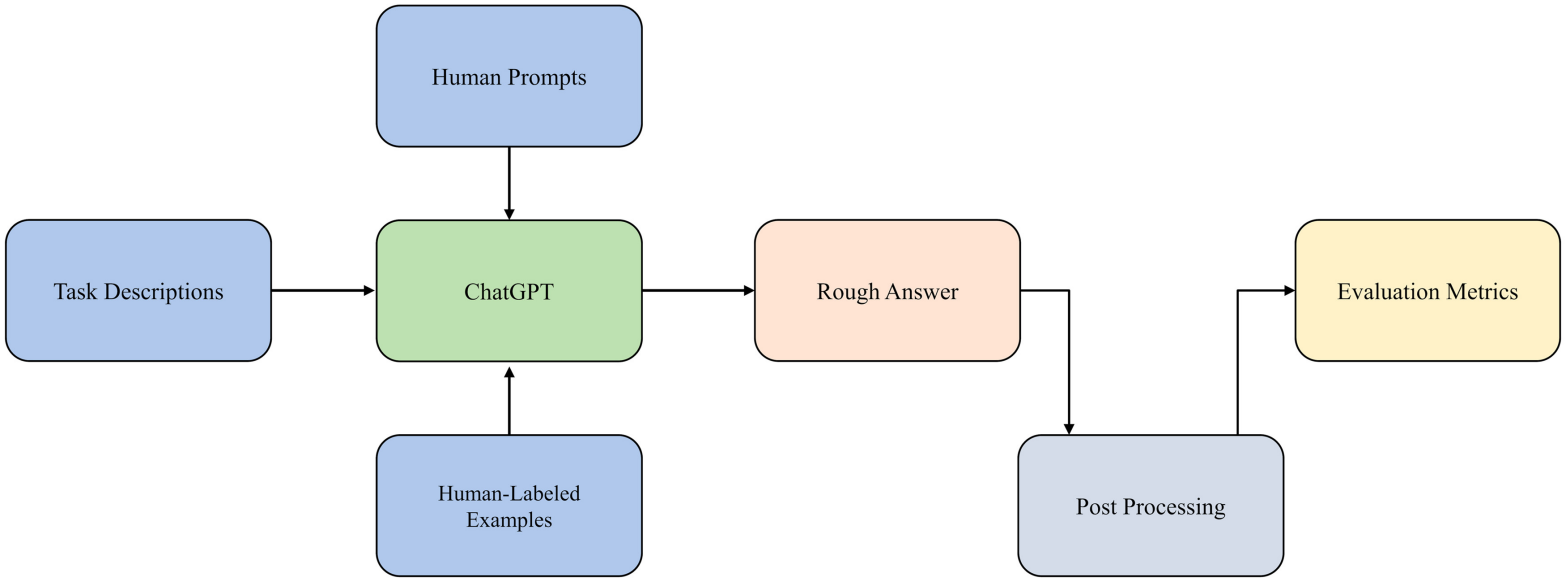
An overview of the workflow for Biomedical NLP using ChatGPT.

Figure 1 provides the workflow of zero-shot biomedical NLP task using ChatGPT.

## 2 Datasets and methods

We applied the pattern proposed in Section ChatGPT for Biomedical NLP to test the performance of ChatGPT on Biomedical NLP tasks. Considering the model accessibility and computation speed, we tested the ChatGPT model built on GPT-3.5 to evaluate the performance on Biomedical NLP tasks. In this section, we will first introduce the benchmark datasets, followed by a description of our tasks and their respective implementation details. Finally, we will present the results of ChatGPT.

### 2.1 Prompt design

In three datasets (ChemProt, DDI, GAD), we tried two types of prompt, one simplified and one complicated. To clarify the design of these two types of prompts, one example in the Biomedical Relation Extraction task (DDI dataset) is shown in Table 1. Additionally, for the explanation of label, one example answer was also provided. Our goal for the design of complex prompt is to provide additional instruction for ChatGPT to better understand the question and the labels of answer.

**Table 1.** Prompts for relation extraction task (DDI dataset).

Prompt	Response	Answer
Text: @*DRUG*$, an anionic-binding resin, has a considerable effect in lowering the rate and extent of @*DRUG*$ bioavailability. Target: You need to identify the relationship between the two @*DRUG*$. Require: you must start with choose one from the [“mechanism,” “effect,” “advice,” “int,” “None”].	None	✗
Text: @*DRUG$* an anionic-binding resin, has a considerable effect in lowering the rate and extent of @*DRUG*$ bioavailability. Target: You need to identify the relationship between the two @*DRUG*$. Require: you must start with choose one from the [“mechanism,” “effect,” “advice,” “int,” “None”], Specific Explanation: mechanism: This type is used to annotate DDIs that are described by their PK mechanism (e.g. Grepafloxacin may inhibit the metabolism of theobromine). effect: This type is used to annotate DDIs describing an effect (e.g. In uninfected volunteers, 46% developed rash while receiving SUSTIVA and clarithromycin) or a PD mechanism (e.g. Chlorthali done may potentiate the action of other antihypertensive drugs). advice: This type is used when a recommendation or advice Regarding a drug interaction is given (e.g. UROXATRAL should not be used in combination with other alpha-blockers). int: This type is used when a DDI appears in the text without providing any additional information (e.g. the interaction of Omeprazole and ketoconazole have been established). You should mark the final category with <>.	< mechanism>	✓

### 2.2 BLURB benchmark

We utilized a comprehensive benchmark dataset for Biomedical NLP, the *Biomedical Language Understanding & Reasoning Benchmark (BLURB)*, which is an extensive collection of biomedical NLP tasks derived from publicly accessible data sources and contains 13 biomedical NLP subsets grouped in six types of tasks. These tasks include NER, evidence-based medical information extraction (PICO), biomedical relation extraction (BRE), sentence similarity, document classification, and QA. An overview of the BLURB datasets can be found in Table 2.

**Table 2.** Overview of the BLURB benchmark.^a^

Dataset	Task	Train	Dev	Test	Evaluation metrics
BC5-chem	NER	5203	5347	5385	F1 entity-level
BC5-disease	NER	4182	4244	4424	F1 entity-level
NCBI-disease	NER	5134	787	960	F1 entity-level
BC2GM	NER	15197	3061	6325	F1 entity-level
JNLPBA	NER	46750	4551	8662	F1 entity-level
EMB PICO	PICO	339167	85321	16364	Macro F1 word-level
ChemProt	BRE	18035	11268	15745	Micro F1
DDI	BRE	25296	2496	5716	Micro F1
GAD	BRE	4261	535	534	Micro F1
BIOSSES	Sentence similarity	64	16	20	Pearson
HoC	Document classification	1295	186	371	Average Micro F1
PubMedQA	QA	450	50	500	Accuracy
BioASQ	QA	670	75	140	Accuracy

a We list the numbers of instances in train, dev, and test, as well as their respective evaluation metrics.

To calculate the overall score for BLURB, the simplest approach would be reporting the average score across all tasks. However, this may be biased by some high-scored tasks. Therefore, we provided both average score per task class which reflects the performance on datasets belonging to the same task type, and the average overall score among all task types.

### 2.3 Biomedical NLP tasks

In order to achieve optimal performance for ChatGPT model across different tasks, specific prompts for various tasks were designed based on the pattern proposed in Section ChatGPT for Biomedical NLP.

#### 2.3.1 Named entity recognition

NER task is a process for identifying and predicting named entities, such as name of chemical substance, disease, gene, and protein, within given input text. Five NER datasets from the BLURB benchmark were investigated, including *BC5-Chemical*, *BC5-Disease*, *NCBI-Disease*, *BC2GM*, and *JNLPBA*. For these datasets, the same splits for train, validation, and test set as utilized by Crichton *et al.* (2017) were employed in current study. BC2GM (Smith *et al.* 2008) is a corpus dataset, which consists of over 20 000 abstracts and full-text articles from the MEDLINE database published during the period 1991–2003. Each document in the corpus was annotated by domain experts with gene names and synonyms. The NER task on the BC2GM dataset requires a predictive model to identify all gene entities mentioned in a text. The BC5-chem and BC5-disease datasets were retrieved from the BioCreative challenge and were respectively designed for NER tasks toward chemical and disease entities. The former dataset contains over 1500 documents with approximately 42 000 chemical annotations, while the latter one contains over 1500 documents with approximately 24 000 disease annotations. The NCBI-disease corpus was created by the National Center for Biotechnology Information (NCBI) for disease recognition tasks in biomedical natural language processing (Doğan *et al.* 2014). It consists of over 793 PubMed abstracts that were manually annotated by domain experts with disease names and their corresponding disease IDs from the Medical Subject Headings (MeSH) vocabulary. The JNLPBA (Joint Workshop on Natural Language Processing in Biomedicine and its Applications) corpus was provided by the JNLPBA conference specifically for gene entity recognition (Collier and Kim 2004). It consists of over 2000 PubMed abstracts, manually annotated by domain experts. These corpora cover a diverse range of biomedical topics, making it a valuable resource for training and evaluating machine learning models for NER tasks. In the BLURB, the annotation format in the corpus was unified for five NER datasets. Specifically, a pair of entity type masks were added before and after the words representing the entity name. For example, the mask “gene* [entity] *gene” was inserted to the text to label the gene entity in the bracket. The disease and chemical entities were masked similarly. In this study, ChatGPT was employed to recognize the entity name in the text without any prior knowledge. The prompt was designed as:

*Paragraph:* <*Paragraph ID*>*—* <*text* >4*Please extract all chemicals/genes/diseases mentioned in the paragraph. Answer with the format “*<*Paragraph ID* >|<*recognized entities*>*”*

#### 2.3.2 PICO

PICO stands for Patient/Population, Intervention, Comparison, and Outcomes. PICO model is used to construct a clinical question. The practice of evidence-based medicine (EBM) aspires to inform healthcare decision using the total relevant evidence (Nye *et al.* 2018). *EBM-NLP* is an biomedical corpus comprising 4993 medical abstracts describing clinical trials, containing spans of token corresponding to three categories, i.e. Populations, Interventions, and Outcomes in the clinical trial. Each P/I/O span is further annotated with more detailed labels, e.g. Age, Sex information, etc. (Huang *et al.* 2006). The test set contains 191 abstracts where 16 364 out of around 54 000 tokens are related to P/I/O categories and others are labeled as “None.” Comparison(C) is not annotated in this corpus. This is like a token-wise multiclassification task as typical classifiers did. But it is inconvenient to ask ChatGPT to classify each word one by one. In practice, we designed prompts similar to the NER tasks for asking ChatGPT to extract all the words related to P/I/O class and the rest of words were attributed as “None.” A natural-language-like prompt was designed as:

*Reference:* <*abstract*>*The reference describe a clinical trial. Which words are about the participants / interventions / outcomes? You can only answer with words or phrase in the reference. If nothing mentioned, answer “None”.*

The PICO task is somehow similar to a NER task but there are still some differences between the tasks. For example, the words annotated as P/I/O can not only be entity names, but also sentences composed of prepositions, adverbs, and even punctuations etc, which describe the target span. As the result is evaluated with macro word-level F1 score, a neural network classifier can make a prediction for each token(word), but it is impractical for ChatGPT, a generative model, to do the task in such a word-wise way. For example, ChatGPT only answers a word for one time even if the word appears several times in the abstract. In order to properly evaluate the performance of ChatGPT, these words were weighted with the number of appearance when counting the confusion matrix, and the punctuations were excluded.

#### 2.3.3 Biomedical relation extraction

Biomedical relation extraction (BRE) task focuses on identifying and extracting relationships between medical entities in input text, such as connections between diseases and drugs, or symptoms and treatments. Formally, let *x* represents a sentence containing two medical entities, $e_{1}$ and $e_{2}$, with *r* being the relation between them. The BRE task can be framed as a classification problem, where the objective is to learn a function $f(x,e_{1},e_{2})\to r$, with *r* belonging to the set of possible relations *R*. This function leverages the context provided by sentence *x* to predict the relation between entities ($e_{1}$, $e_{2}$). The performance of BRE models is generally assessed using standard classification metrics, such as confusion matrix-based Precision, Recall, F1-score, etc. We evaluated the performance of ChatGPT on three biomedical datasets: ChemProt, DDI, and GAD. To assess ChatGPT’s ability in the BRE task, e.g. we crafted a prompt (for the GAD Dataset) as following:

*“Does the reference indicate a relationship between the @DISEASE$ and the @GENE$ without specifying the exact disease and gene? Response with “yes” or “no”.”* By doing in this way, it allowed us to gauge ChatGPT’s effectiveness in recognizing and extracting relationships between medical entities within the context of biomedical text.

As indicated by its name, ChemProt is a dataset containing around 700 000 unique chemicals, 3000 proteins and 2 000 000 interactions overall from around 2500 documents. All the interactions are grouped into 10 groups according to biological semantic classes. A five-group-subset was used as the test set. The five groups in the test set include: (i) upregulator | activator | indirect upregulator, (ii) downregulator | inhibitor | indirect downregulator, (iii) agonist | agonist-activator | agonist-inhibitor, (iv) antagonist, and (v) substrate | product of | substrate product of. There are even more groups in the train set and validation set. Besides, unrelated chemical substance and protein pairs were labeled as “None” to enrich the dataset. Clearly, domain knowledge is required to help understand what the exact relation means and might be missing in general LLMs like ChatGPT. To overcome the difficulty, we test ChatGPT by adding one sample of the validation set for each group into the prompt, in another word, with the one-shot learning manner.

The Drug–Drug Interaction corpus (Herrero-Zazo *et al.* 2013) was created to facilitate research on pharmaceutical information extraction, with a particular focus on pharmacovigilance. It contains sentence-level annotation of drug-drug interactions on PubMed abstracts.

Gene-disease associations database (GAD) (Bravo *et al.* 2015) set is a collection of around 5000 published gene/disease associations. The gene name and disease name in the document are recognized and masked. Here, the label indicates whether the document implies an association between the gene and the disease as a binary classification task. Different from ChemProt, the relations were not strictly defined with a biology terminology and could be ambiguous sometimes. 534 sentences were used as the test set.

#### 2.3.4 Sentence similarity

The Sentence Similarity task involves predicting a similarity score based on the likeness of a given pair of sentences. The BLURB benchmark contains the *BIOSSES* dataset consisting of 100 pairs of sentence from Text Analysis Conference(TAC) Biomedical Summarization Track (Soğancıoğlu *et al.* 2017). The train, validation, and test splits were the same with the ones used before (Peng *et al.* 2019) and we tested ChatGPT on a test set of 20 pairs. The score is in {0, 1, 2, 3, 4}. The definition is declared in the Table 3. Each sample was scored by five annotators and the average score was used as the ground truth, leading to a regression-like task. The prompt is designed as: *What is the similarity score between the* <*sentence1*>*and the* <*sentence2*>*? Response with float ranging from 0 (no relation) to 4 (equivalent)?*

**Table 3.** Definition of the scores in the BIOSSES dataset.

Score	Comment
0	The two sentences are on different topics.
1	The two sentences are not equivalent, but they are on the same topic.
2	The two sentences are not equivalent, but share some details.
3	The two sentences are roughly equivalent, but some import information differs/missing.
4	The two sentences are completely or mostly equivalent, as they mean the same thing.

#### 2.3.5 Document classification

Document Classification is a procedure of assigning one or more predefined labels to a document. Evaluation for this task was done at the document level, i.e. aggregating labels across all sentences within a document. We utilized the HoC dataset from the BLURB benchmark, which was curated by Baker *et al.* (2016) and employed the same splits of train, validation, and test set. We have designed the following prompt to enable ChatGPT to carry out the document classification task:

“*document:* <*text*>*; target: The correct category for this document is ? You must choose from the given list of answer categories (introduce what each category is …)*.”

#### 2.3.6 Question answering

The QA task refers to predicting answers under the given context, in which the first sentence is question. Answers are either two labels (yes/no) or three labels (yes/maybe/no). We utilized the PubMedQA (Jin *et al.* 2019a) and BioASQ datasets for evaluation. For both datasets, the original train, validation, and test splits within the BLURB benchmark were used.

For evaluation of ChatGPT on PubMedQA and BioAS, we simply designed the following prompt: *“question:* <*text*>*; context:* <*text*>*; answer:* <*text*>*; target: the answer to the question given the context is (yes or no)?”*

### 2.4 Baseline models

We selected three baseline models that are SOTA on the BLURB benchmark for comparison with ChatGPT, i.e. **PubmedBERT**, **BioLinkBERT-Base**, and **BioLinkBERT-Large**. All the models were built on the BERT architecture. **PubmedBERT** (Gu *et al.* 2021) was pretrained on PubMed corpus while **BioLinkBERT-Base** (Yasunaga *et al.* 2022) was pretrained on PubMed with citation links. The **BioLinkBERT-Large** model was specifically pretrained on a large corpus of biomedical literature and clinical notes, which allow to capture the complex terminology and domain-specific knowledge required for biomedical NLP tasks. It contains over 335 million parameters, making it one of the largest pretrained models in the biomedical domain.

## 3 Results and discussions

We tested the performance of ChatGPT with engineered prompts as mentioned in previous sections and altogether, six types of biomedical text mining task (NER, PICO, BRE, Sentence Similarity, Document Classification, and QA) were explored.

Table 4 shows the performance of ChatGPT and baseline models on BLURB benchmark. Although, in general, ChatGPT got a BLURB score of 59.46 which is significantly worse than the SOTA baselines, there are still interesting conclusions can be drawn for ChatGPT. On the other hand, we should bear in mind that ChatGPT was trained as a general language model, while the baselines are models particularly trained on biomedical corpus.

**Table 4.** Performance on BLURB benchmark.^a^

	PubMedBERT	BioLinkBERT-Base	BioLinkBERT-Large	ChatGPT
Named entity recognition	86.27	86.19	**86.89**	48.27
BC5-chem (Li *et al*. 2016)	93.33	93.75	94.04	60.30
BC5-disease (Li *et al*. 2016)	85.62	86.10	86.39	51.77
NCBI-disease (Dogan *et al*. 2014)	87.82	88.18	88.76	50.49
BC2GM (Smith *et al*. 2008)	84.52	84.90	85.18	37.54
JNLPBA (Collier and Kim 2004)	80.06	79.03	80.06	41.25
PICO extraction	73.38	73.97	**74.19**	55.59
EBM PICO (Nye *et al*. 2018)	73.38	73.97	74.19	55.59
Relation extraction	80.65	81.56	**82.74**	46.08
ChemProt (Krallinger *et al*. 2017)	77.24	77.57	79.98	34.16^b^
DDI (Herrero-Zazo *et al*. 2013)	82.36	82.72	83.35	51.62
GAD (Becker *et al*. 2004)	82.34	84.39	84.90	52.43
Sentence similarity	92.30	93.25	**93.63**	43.75
BIOSSES (Soğancıoğlu *et al*. 2017)	92.30	93.25	93.63	43.75
Document classification	82.34	84.39	**84.90**	51.22
HoC (Baker *et al*. 2016)	82.34	84.39	84.90	51.22
Question answering	71.70	80.82	**83.50**	82.51
PubMedQA	55.84	70.20	72.18	**76.45**
BioASQ (Nentidis *et al*. 2020b)	87.56	91.43	**94.82**	88.57
BLURB score	81.10	83.39	**84.30**	58.50

a We list the overall BLURB Score and the score for each task in **Bold**.

b We also tested the dataset in a one-shot manner and the corresponding score is 48.64%.

Among all types of tasks, QA task is the only type of task that ChatGPT is comparative to the baselines. In this case, ChatGPT (82.5) outperforms PubMedBERT (71.7) and BioLinkBERT-Base (80.8) and is very close to the BioLinkBERT-Large (83.5). In particular on the PubMedQA dataset, ChatGPT exceeded the baselines significantly and the score is close to the human performance of 78.2% (Jin *et al.* 2019a) and the SOTA score of 79.6% (He *et al.* 2022). The good performance of ChatGPT on QA task might be related to the fact that ChatGPT is designed as a chatbot, and the capability is carried over pretty well into the biomedical domain.

The NER tasks in BLURB are to identify entities of chemical substance, disease and gene name. The recognition accuracy of ChatGPT among various datasets is, from high to low, chemicals (BC5-chem) > diseases (BC5-disease and NCBI-disease) > genes (BC2GM and JNLPBA), which is consistent with the baselines (Table 5). This trend reflects that disease and gene name have higher intrinsic complexity than chemical name. We attribute the poor performance of ChatGPT to the missing of supervised training and lack of training data in biomedical field. As far as we know, ChatGPT was trained mainly on the data of web sites, social media posts, books, and articles. But biomedical entities, especially terminologies, are uncommon in the daily usage. It is probably explainable that ChatGPT does not understand well these texts which need more domain knowledge to interpret.

As introduced in the Section 6, PICO task is similar to NER. ChatGPT performs worse than the baselines but the gap is smaller comparing to NER tasks (Table 6). PICO task was assumed to be easier since many of the target words/sentences are commonly used in daily life and easy to understand. One possible reason for the poor performance is that ChatGPT may miss the short sentences or phrases while can successfully extract the long ones. Among following phrases and sentences labeled as P class in a document “treated hypertensive patients,” “hypertensive patients receiving drug treatment,” “hypertensives on chronic, stable antihypertensive therapy,” “people with one or more cardiovascular risk factors,” “hypertensives under treatment,” “Fifteen Italian hypertension units studied 142 hypertensive patients (76 men, 66 women; mean age 59 ± 5.9 years) treated with different antihypertensive drugs,” ChatGPT failed to recognize those short phrases/sentences as P class and only labeled the last long sentence correctly.

Relation extraction tasks require a model to be able to identify the relation of a pair of entities masked in the text. As it was mentioned earlier, for the three datasets (ChemProt, DDI, GAD) of relation extraction, two different prompts were designed. The performance of these two prompt types was listed in Supplementary Table S1. Overall, complex prompts achieved better performance than simple ones. For the example shown in Table 1, the simple prompt triggered a wrong answer “None” as the relationship, while, for the complex prompt, the correct answer “mechanism” was obtained. The results of complex prompt were used as the final results of Table 4. For DDI and GAD datasets, whose format is similar to QA tasks requiring the model output to be a simple “yes” or “no,” ChatGPT performed poorly. ChemProt set is more complex due to the requirement of grouped relation and ChatGPT got even lower score than other tasks. A straightforward guess is that the so-called “relation” is not that clear. It is hard for ChatGPT to understand what the relation mentioned in our prompt refers to. To validate the guess, we tested ChatGPT on ChemProt in one-shot manner, in which one sample for each relation group was provided. The one-shot method greatly improved the score from 34.16% to 48.64%. Another thing we noticed from the results was that ChatGPT tended to be confused by other words in the text and often assigned relation labels to entity pairs which are actually unrelated. The original ChemProt data contains only 3458 test samples where the entity pairs are all related. While in BLURB benchmark set, this set was augmented with 15 745 negative pairs. It was found that false positive rate (FPR) is as high as 75%. We tested ChatGPT on the original ChemProt data and the F1-score is 79.93%. Through these experiments, we expect that ChatGPT still has room to do a better job on these tasks with more carefully designed prompts, e.g. supplying more instructions about the relation that the dataset concerns and add more shots.

The document classification task is quite challenge for ChatGPT. On one hand, the number of the answer category is uncertain, it may be an empty category, it may be one of the categories, or it may be multiple categories. On the other hand, this few shot learning scenario is not friendly for ChatGPT, as it is really difficult to understand the labels without enough domain knowledge. It can be seen from Table 4 that on the HoC dataset, ChatGPT only obtained an F1 value of 51.22%, which is much worse than BERT based models, indicating that the performance of ChatGPT in processing medical text classification tasks with few samples is still far from optimal.

Sentence similarity is also a difficult case for ChatGPT with zero-shot. Different from other tasks, the similarity defined on the BIOSSES data is quite subjective and the similarity score could be ambiguous. The Y variable is the average score from five annotators and the human opinions are always diverse. The score deviation of a certain pair of sentences could be up to two. The Pearson coefficient between individual annotations and the ground truth is only 0.5. The baselines got a high score due to the fine-tune process. ChatGPT may work better on this task if we fed some samples from the train set within the prompt. As we focused mainly on the zero-shot method and tried to evaluate the overall capacity of the ChatGPT, we did not test this strategy for this small dataset with only 100 pairs of sentences.

**Table 5.** Metrics for five NER tasks with BLURB benchmark datasets.

NER Task	F1-score	Recall	Precision
BC5-disease	0.52	0.59	0.46
BC2GM	0.38	0.46	0.32
BC5-chem	0.60	0.76	0.50
NCBI-disease	0.50	0.51	0.50
JNLPBA	0.41	0.55	0.33

**Table 6.** Performance of ChatGPT on EBM PICO task.^a^

Metrics	P	I	O	Macro average
*N*	4050	3102	7033	
Precision	73.78	57.76	48.64	
Recall	49.95	65.96	42.92	
F1-score	59.57	61.59	45.60	55.59

a Annotated punctions are excluded.

## 4 Conclusion

Based on our experiments, the ChatGPT built on the early version of GPT-3.5 performed poorly on several biomedical NLP benchmark datasets. The biomedical domain is clearly a challenging professional field to deal with for a general LLM running in the zero or few shot scenario. Another common problem is that ChatGPT is a generative model while most benchmark sets are designed for supervised models, requiring a structured prediction. SOTA language models are usually fine-tuned in a supervised manner based on a pretrained large model. Though we can add instructions in the prompt to constrain the output of the ChatGPT, there are still chances that the ChatGPT output does not follow the expected format. Having said that, the superior version GPT-4 has recently been released and demonstrated better ability of natural language understanding and reasoning. We are looking forward to test newer version of ChatGPT on professional NLP tasks to explore the potentiality of LLMs.

## Supplementary Material

btad557_Supplementary_Data
