# Supplementary material for: An extensive benchmark study on biomedical text generation and mining with ChatGPT
Source: Bioinformatics. 2023 Sep 8;39(9):btad557. doi: 10.1093/bioinformatics/btad557 (PMC10562950; doi:10.1093/bioinformatics/btad557)
Supplement: btad557_Supplementary_Data [file btad557_supplementary_data.pdf]

**Table S1.** Performance of ChatGPT on biomedical-NLP tasks with different prompt.

| Dataset | Prompt | Metrics |
|---|---|---|
| ChemPort | According to the reference: <text>, What is the relation between ent1 and ent2? Answer with one word within ’activator’, ’inhibitor’, ’agonist’, ’antagonist’, ’substrate’ or ’None’. | 34.16% |
| | According to the reference <text>, what is the relation between the @CHEMICAL$ and @GENE$ ignoring what they exactly are? The relation between a chemical and a gene can be [”activator”, ”inhibitor”, ”agonist”, ”antagonist”, ”substrate”, ”production”]. Here are some examples: {1 example for each label According to the reference}. Answer in one words within [”activator”, ”inhibitor”, ”agonist”, ”antagonist”, ”substrate”, ”production”]. If the relation can not be determined with the reference or they have no relation, answer ”False”. | 48.54% |
| GAD | Reference: <text> Dose the reference reflect a relation between the @DISEASE$ and the @GENE$ without inferring what the exact disease and gene are? Answer with ”yes” or ”no”.’ | 47.19% |
| | Reference: <text> Dose the reference reflect a relation between the @DISEASE$ and the @GENE$ without inferring what the exact disease and gene are? Answer with ”yes” or ”no”. | 52.43% |
| DDI | Text: <text> Target: you need to identify the relationship between the two @DRUG$. Require: you must answer within [’mechanism’, ’effect’, ’advice’, ’int’, ’None’] | 45.80% |
| | Target: you need to identify the relationship between the two @DRUG$. Require: you must answer within [’mechanism’, ’effect’, ’advice’, ’int’, ’None’] <mechanism>: This type is used to annotate DDIs that are described by their PK mechanism (e.g. Grepafloxacin may inhibit the metabolism of theobromine). <effect>: This type is used to annotate DDIs describing an effect (e.g. In uninfected volunteers, 46% developed rash while receiving SUSTIVA and clarithromycin) or a PD mechanism (e.g. Chlorthalidone may potentiate the action of other antihypertensive drugs). <advice>: This type is used when a recommendation or advice regarding a drug interaction is given (e.g. UROXATRAL should not be used in combination with other alpha-blockers). <int>: This type is used when a DDI appears in the text without providing any additional information (e.g. The interaction of omeprazole and ketoconazole has been established). <none>: The text does not mention the relationship between the two drugs. | 51.62% |
